# A Novel Long Noncoding RNA, lncR-125b, Promotes the Differentiation of Goat Skeletal Muscle Satellite Cells by Sponging miR-125b

**DOI:** 10.3389/fgene.2019.01171

**Published:** 2019-11-15

**Authors:** Siyuan Zhan, Chenyu Qin, DanDan Li, Wei Zhao, Lu Nie, Jiaxue Cao, Jiazhong Guo, Tao Zhong, Linjie Wang, Li Li, Hongping Zhang

**Affiliations:** ^1^Farm Animal Genetic Resources Exploration and Innovation Key Laboratory of Sichuan Province, Sichuan Agricultural University, Chengdu, China; ^2^College of Animal Science and Technology, Sichuan Agricultural University, Chengdu, China

**Keywords:** long noncoding RNA, competing endogenous RNA, microRNA, muscle differentiation, goat

## Abstract

Long noncoding RNAs (lncRNAs) have emerged as essential regulators of skeletal myogenesis, but few myogenesis-associated lncRNAs have been identified and our understanding of their regulatory mechanisms remains limited, particularly in goat. Here, we identified a novel lncRNA, TCONS_00006810 (named lncR-125b), from our previous lncRNA sequencing data on fetal (45, 60, and 105 days of gestation, three biological replicates for each point) and postnatal (3 days after birth, n = 3) goat skeletal muscle, and found that it is highly expressed in skeletal muscle and gradually upregulated during skeletal muscle satellite cell (SMSC) differentiation in goat. Notably, overexpression of lncR-125b accelerated the expression of myogenic differentiation 1 (MyoD 1) and myogenin (MyoG), and the formation of myotubes, and knockdown of lncR-125b showed opposite effects in SMSCs. Results of dual-luciferase assay and quantitative real-time polymerase chain reaction revealed that lncR-125b acts as a molecular sponge for miR-125b and that insulin-like growth factor 2 (IGF2), a critical regulator of skeletal myogenesis, is a direct target gene of miR-125b. Further analyses showed that lncR-125b negatively regulates miR-125b expression and positively regulates IGF2 expression in SMSCs. Mechanistically, lncR-125b promotes SMSC differentiation by functioning as a competing endogenous RNA (ceRNA) for miR-125b to control IGF2 expression. These findings identify lncR-125b as a novel noncoding regulator of muscle cell differentiation and skeletal muscle development in goat.

## Introduction

Myogenesis is a complex biological process that involves the sequential expression of a number of transcription factors including Paired box 3 (Pax3) and Pax7, followed by the expression of myogenic regulatory factors (MRFs), such as Myogenic Factor 5 (Myf5), MyoD, MyoG, and Myogenic Factor 6 (Myf6) (Berkes and Tapscott, 2005; Braun and Gautel, 2011; Moncaut et al., 2013), and myocyte enhancer factor-2 (MEF2) (Naya and Olson, 1999; Estrella et al., 2015). These factors orchestrate the activities of a variety of muscle-related genes, resulting in the strict control of myogenesis. Previous studies have demonstrated the crucial role of microRNAs (miRNAs) in the control of skeletal myogenesis (Erriquez et al., 2013; Diniz and Wang, 2016; Kopantseva and Belyavsky, 2016). It is now becoming evident that lncRNAs are important players in the processes of skeletal muscle growth and development. For example, steroid receptor RNA activator (SRA) was identified as the first example of an lncRNA involved in myogenesis by upregulating MyoD, the master transcription factor involved in muscle differentiation (Caretti et al., 2006; Hube et al., 2011). Another established lncRNA, *Dum* (Developmental pluripotency associated 2 Upstream binding Muscle lncRNA), silences its neighboring gene, *Dppa2* (Developmental pluripotency associated 2), *in cis* through the recruitment of multiple DNA methyltransferases to its promoter region, leading to *Dppa2* silencing by hypermethylation, thus promoting myogenesis (Wang et al., 2015). In addition, Linc-RAM (Linc-RNA Activator of Myogenesis) acts as a regulatory lncRNA directly interacting with MyoD to facilitate assembly of the MyoD-Baf60c-Brg1 complex and then promotes myogenic differentiation (Yu et al., 2017). It has been reported that an lncRNA, lncYYW, can promote bovine myoblast proliferation by regulating GH1 expression (Yue et al., 2017). Moreover, lncRNAs might encode latent functional polypeptides that are involved in regulating muscle performance (Anderson et al., 2015; Nelson et al., 2016; Matsumoto et al., 2017). These studies indicate the importance of lncRNAs in muscle biology.

Recent studies have revealed that lncRNAs can act as competing endogenous RNAs (ceRNAs) in the regulation of muscle formation (Cesana et al., 2011; Sun et al., 2016; Wang et al., 2016; Jin et al., 2017; Zhu et al., 2017; Liang et al., 2018). ceRNAs can impair miRNA activity by acting as molecular sponges for miRNAs, thereby upregulating miRNA target gene expression (Salmena et al., 2011; Kartha and Subramanian, 2014; Tay et al., 2014; Thomson and Dinger, 2016). For instance, linc-MD1 upregulates the expression of myocyte enhancer factor 2C (MEF2C) and mastermind-like transcriptional coactivator 1 (MAML1), which activate muscle-specific gene expression by competitively binding miR-133 and miR-135 and govern muscle differentiation in mouse and human myoblasts (Cesana et al., 2011). Myogenesis-associated lncRNA (lnc-mg), also a ceRNA, was recently shown to be a skeletal muscle-enriched lncRNA that enhances myogenesis *in vitro* and *in vivo* (Zhu et al., 2017). H19 acts as a ceRNA, “sponging” let-7 (Kallen et al., 2013), which leads to the derepression of HMGA2 and IGF2BP2, two essential factors in skeletal muscle satellite cell (SMSC) proliferation (Li et al., 2012b). In addition, metastasis-associated lung adenocarcinoma transcript 1 (Malat1) contains a functional miR-133 target site and can regulate myocyte differentiation by competing for miR-133 (Han et al., 2015). A recent study found that lncR-133b promotes bovine SMSC proliferation in growth medium (GM) and represses SMSC differentiation in differentiation medium (DM) by regulating the expression of the target genes of miR-133b (Jin et al., 2017).

As animals that are economically important worldwide, domestic goats (*Capra hircus*) are raised mainly for meat production. Thus, it is important to reveal the molecular mechanisms behind their skeletal muscle formation and development. Although reports about muscle-related lncRNAs in humans, mice, and cattle have been published, no studies of muscle-related lncRNAs in goat have been reported to the best of our knowledge. Previously we studied the lncRNA transcriptome of fetal and postnatal goat skeletal muscle and identified some lncRNAs that may be involved in muscle development (Zhan et al., 2016). In this study, we identified a new functional lncRNA (lncR-125b) in goat. lncR-125b is highly expressed in *longissimus thoracis et lumborum* (LTL) muscle and induced during SMSC differentiation. Our study further reveals that lncR-125b promotes the differentiation of goat skeletal muscle by functioning as a ceRNA whereby it sequesters miR-125b, thereby augmenting the expression of the miR-125b target gene, IGF2, a crucial regulator of skeletal myogenesis.

## Materials and Methods

### Animals and Tissue Collection

The animals (female, n = 16) used here were Jianzhou big-eared goats from Jianyang Dageda farm (Chengdu, Sichuan, China). Generally, all of the pregnant ewes (healthy, 2–3 years old) were housed in a free stall and fed a standard diet (forage to concentrate ratio, 65:35), twice (06:30–08:30 and 16:00–18:00) daily, and water provided *ad libitum*. Goat fetuses (female, n = 3) were randomly chosen at 60 days of gestation, humanely collected by caesarean section, and sacrificed. A total of six tissues, namely, heart, liver, spleen, lung, kidney, and LTL muscle, were collected and snap-frozen in liquid nitrogen for RNA extraction.

### SMSC Isolation, Culture, and Transfection

The SMSCs used here were successfully isolated from LTL muscles of neonatal goats in our laboratory, as described previously (Li et al., 2015; Zhao et al., 2018). In brief, after a quick washing step with sterile phosphate-buffered saline (PBS, Hyclone, Logan, UT, USA), sampled LTL muscles were minced and digested with 0.2% pronase (Sigma, St. Louis, MO, USA) and placed in a water bath at 37°C for 60 min, followed by centrifugation at 1,500 ×g for 6 min, after which the obtained pellet was retained. After washes with PBS (two times), the pellets were suspended in Dulbecco’s modified Eagle’s medium (DMEM/high glucose; Hyclone) supplemented with 15% fetal bovine serum (FBS, Gibco, Grand Island, NY, USA), filtered through a 70-µm mesh sieve (BD, Franklin Lakes, NJ, USA), and then centrifuged at 800 ×g for 5 min to isolate the SMSCs. The isolated SMSCs were purified using a Percoll gradient (90%, 40%, and 20%) (Sigma) and centrifuged at 1,800 ×g for 50 min to enrich the SMSCs in the Percoll interface between 40% and 90%. Finally, the purified SMSCs were qualified by direct immunostaining with Pax7 (Paired box 7, rabbit anti-Pax7, 1:100 dilution; Boster, Wuhan, Hubei, China), a critical marker for SMSCs. Those Pax7^+^ SMSCs were kept in liquid nitrogen for subsequent experiments.

In general, SMSCs were seeded in 6-well (∼2 × 10^4^ cells per well) or 12-well (∼1 × 10^4^ cells per well) plates and cultured in GM containing DMEM supplemented with 15% FBS in a 5% CO_2_ atmosphere at 37°C. To induce differentiation, GM was replaced by DM containing 2% horse serum (98% DMEM high glucose + 2% HS; Gibco) when SMSCs reached 80%–90% confluence. The medium was replaced by a fresh one every 2 days.

For gain/loss-of-function study, GM was replaced with DMEM containing 15% FBS when cells were at 80%–90% confluence; then, cells were transfected using Lipofectamine 2000 reagent (Invitrogen, Carlsbad, CA, USA) with siRNA (siRNA-lncR-125b, siRNA-IGF2), or overexpression plasmid (pEGFP-lncR-125b, pEGFP-IGF2) and miR-125b mimic or inhibitor (Ribobio, Guangzhou, Guangdong, China) at the indicated concentrations, in accordance with the manufacturer’s instructions. Six hours later, GM was replaced by DM. The transfected cells were harvested at 48 (for qRT-PCR) or 72 h (for western blotting), and subjected to immunofluorescent staining on the seventh day after differentiation.

### RNA Extraction and qRT-PCR

Total RNA was extracted from tissues or cells using TRIzol Reagent (Invitrogen), in accordance with the manufacturer’s instructions. After rough qualification of the degree of degradation and contamination using 1.5% agarose gel electrophoresis, and of the concentration by utilizing a NanoDrop 2000 Spectrophotometer (Thermo-Fisher Scientific, Waltham, MA, USA), the total RNAs (∼1 µg) were reverse-transcribed into cDNA using PrimeScriptTM RT Reagent Kit with gDNA Eraser (Takara, Kusatsu, Shiga, Japan) for mRNA detection, or Mir-X^™^ miRNA First-Strand Synthesis Kit (Takara) for miRNA assay. Using these cDNAs as templates, the expression levels of mRNA, lncRNA, and miRNA were accurately quantified by qRT-PCR in a Bio-Rad CFX96 system (Bio-Rad, Hercules, CA, USA) with SYBR Premix Ex TaqTM II or Mir-X^™^ miRNA qRT-PCR SYBR^®^ Kit (Takara), in accordance with the manufacturer’s protocols. Each reaction (10 µl) contained 5 µl of SYBR, 0.4 µl each of 10 µM sense and antisense primers, 0.8 µl of normalized template cDNA (∼2 µg/µl), and 3.4 µl of sterile water. Amplification conditions were 95°C for 2 min, and then 40 cycles of 10 s at 95°C and 30 s at the annealing temperature (Tm) listed in **Table S1**. Melting curve analysis was performed from 65°C to 95°C with increments of 0.5°C per second. Each experiment was performed in triplicate and repeated three times. The expression levels of mRNAs and lncRNAs were normalized to the housekeeping genes *ACTB*, *YWHAZ*, and *HPRT1* (nine housekeeping genes were used to test expression stability under the experimental conditions). The expression level of miRNA was normalized to U6 snRNA. Relative expression levels were calculated using the 2^-ΔΔCt^ method (Livak and Schmittgen, 2001). The primers are detailed in **Table S1**.

### Plasmid Construction and RNA Oligonucleotides

The genomic fragment of lncR-125b (257 bp) or IGF2 (650 bp) containing an miR-125b binding site was amplified from skeletal muscle cDNA and inserted into a pEGFP-N1 vector (Promega, Madison, WI, USA) using *HindIII* and *BamHI* restriction enzymes to obtain their expression plasmids. Primers used for plasmid construction are listed in **Table S2**. siRNAs specifically targeting lncR-125b or IGF2, miR-125b mimic, and miR-125b inhibitor were purchased from RiboBio company (Guangzhou, Guangdong, China), with nonspecific siRNA sequences as a negative control. DNA sequences of all constructs were confirmed by Sanger sequencing analysis.

### Dual-Luciferase Reporter Assays

The fragments of lncR-125b and IGF2 containing an miR-125b binding site were amplified and subcloned into the *XhoI* and *NotI* sites of the psiCHECK-2 vector (Promega), named lncR-125b-wild and IGF2-wild. The mutant derivatives (lncR-125b-mut, IGF2-mut), accessed by altering the miR-125b binding sites and generated using MutanBEST Kit (Takara), were also cloned into the psiCHECK-2 vector. Mutant plasmids were generated by changing the binding site of miR-125b from CTCAGGG to GTCCATA (lncR-125b-mut), or from TCAGGGA to CACATAA (IGF2-mut). The sequences of wild-type and mutated sequences are presented in **Figure S1**. Primers used for plasmid construction are listed in **Table S2**. For the luciferase reporter assays, SMSCs (∼1 × 10^4^ cells per well) were cultured in GM in 24-well plates, with conditions similar to those for SMSCs, and transfected when cells reached 80%–90% confluence. GM was completely replaced by DM at 6 h post-transfection. The transfected cells were harvested and lysed with Cell Lysis Buffer (NEB, Ipswich, MA, USA) and luciferase activities were measured 48 h after differentiation using a Dual-Luciferase^®^ Reporter Assay System (Promega).

### Immunofluorescence Analyses

SMSCs (seeded at ∼2 × 10^4^ cells per dish) in 3.5-cm Petri dishes cultured in DM for 5 or 7 days were washed three times with ice-precooled PBS; after removing the culture medium, they were fixed with 4% paraformaldehyde for 15 min at room temperature, washed three times again with 1 ml of PBS after removing the paraformaldehyde, permeabilized with 1 ml of 0.5% Triton X-100 at 4°C for 10 min, washed with PBS three times, and blocked with 1 ml of 2% bovine serum albumin (BSA) for 30 min at 37°C. The cells were then incubated with anti-mouse Myosin Heavy Chain (MyHC) (1:150; Abcam, Cambridge Science Park, Cambridge, UK) overnight at 4°C with gentle shaking, washed three times with PBS (5 min each), and subsequently incubated with secondary antibodies Cy3_IgG (H + L) (1:150, Cell Signaling Technology, Beverly, MA, USA) at 37°C for 2 h, followed by washing with PBS again. Finally, the cells were stained with 0.05 µg/ml DAPI (4′,6′-diamidino-2-phenylindole; Invitrogen) at 37°C for 10 min in a humidified dark chamber. After washing three times again with 1 ml of PBS, images were obtained with a fluorescent microscope (Leica, Wetzlar, Germany). ImageJ software (National Institutes of Health, Bethesda, MD, USA) was employed to count cells in each photo, including the total number of nuclei (on DAPI channel), number of nuclei surrounded by MyHC signal, and number of MyHC+ myotubes containing three or more nuclei. The proportion of MyHC-positive cells was then calculated as the proportion of nuclei surrounded by MyHC signal relative to the total number of nuclei. For each treatment, at least three samples were independently used and five areas per sample were randomly selected for analysis.

### Nuclear and Cytoplasmic RNA Fractionation

Nuclear/cytoplasmic fractionation of SMSCs was performed in accordance with the manufacturer’s instructions using cytoplasmic/nuclear RNA Purification Kit (Norgen Biotek, Thorold, ON, Canada). Briefly, SMSCs (seeded at ∼2 × 10^5^ cells per dish) cultured on a 10-cm culture dish at 0 and 7 days of differentiation were washed twice with cold PBS, lysed in hypotonic buffer (10 mM Tris pH 8.0, 1 mM EDTA), and then centrifuged at 500 ×g for 5 min to separate nuclei and debris. Thereafter, total RNAs from both the supernatant (cytosolic fraction) and the pellet (nuclei) were separately extracted using RNAiso Plus reagent (TaKaRa), then reverse-transcribed into cDNA using PrimeScript^™^ RT Reagent Kit with gDNA Eraser (Takara) for quantifying the RNA levels using qRT-PCR. GAPDH and U6 acted as cytoplasmic and nuclear controls, respectively. All procedures were performed at 4°C under RNase-free conditions.

### Western Blot Analysis

To measure the protein levels of IGF2, the total proteins from cultured SMSCs were extracted using the Total Protein Extraction Kit (Beyotime, Shanghai, China) and quantified by employing BCA Protein Quantitation Kit (Beyotime), in accordance with the manufacturer’s instructions. In brief, with ∼20 µg of protein per sample, western blotting was performed through separating protein by 10% sodium dodecyl sulfate-polyacrylamide gel electrophoresis (SDS-PAGE), transferring to a PVDF membrane (Millipore, Bedford, MA, USA), blocking with Blocking Buffer (Beyotime) for 1 h at room temperature, and then incubating the membrane sequentially with the primary anti-mouse IGF2 (1:1,000) (Abcam). After three washes in PBST (0.1% Tween 20 in PBS), the membranes were incubated in secondary antibody (Beyotime) conjugated with horseradish peroxidase (HRP, 1:4,000) for 1.5 h, followed by three washes in PBST. Eventually, we measured the enhanced chemiluminescence signal (ECL) using BeyoECL Plus (Beyotime) and performed visualization using the ChemiDoc XRS+ system (Bio-Rad, with the GAPDH antibody (1:2,000, mouse) (Abcam) as a loading control.

### Statistical Analyses

The data are presented as the mean value ± SEM (standard error of the mean) based on at least three biological replicates. All data were assessed for statistical significance using one-way analysis of variance (ANOVA) and Student’s *t*-test analyses in SAS software version 9.0 (SAS, Cary, NC, USA) with the significance level set at corrected p-value < 0.05.

## Results

### Identification and Expression of lncR-125b

**Figure 1:**
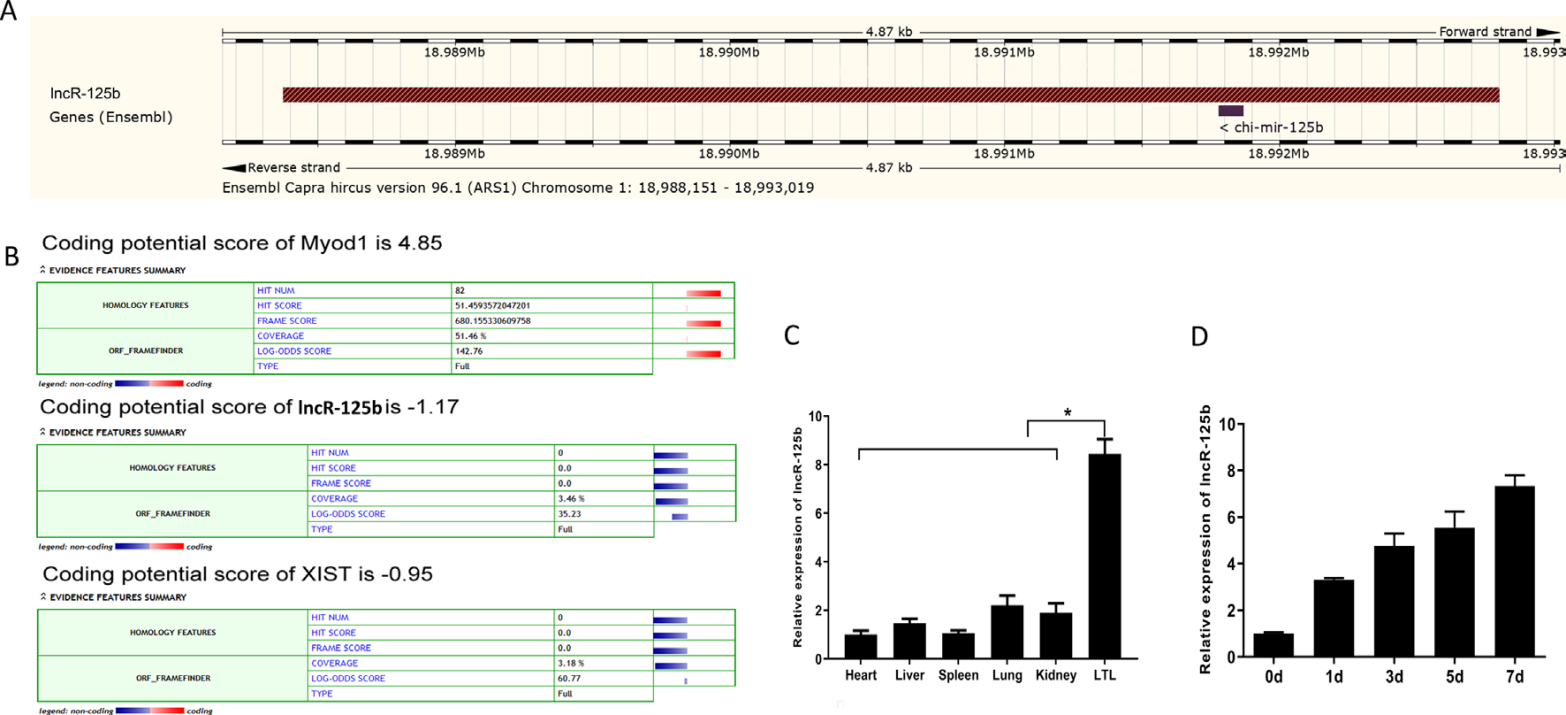
Characterization and expression profile of lncR-125b. **(A)** Genomic locus of lncR-125b in goat chromosome. **(B)** Bioinformatics analysis of the coding capability of lncR-125b, and Myod1 was identified to code for protein, while XIST was identified as noncoding RNA. **(C)** Real-time qPCR analysis of lncR-125b expression in heart, liver, spleen, lung, kidney, and *longissimus thoracis et lumborum* (LTL) muscle of goat. Mean values ± SEM, n = 3, *P < 0.05. **(D)** Real-time qPCR analysis of lncR-125b expression during goat SMSC differentiation (GM and DM for 1, 3, 5, and 7 days). Mean values ± SEM, n = 3.

Our previous study found that several lncRNAs, including TCONS_00006810 (named lncR-125b), exhibit differential expression in fetal and postnatal muscle tissue of goats (Zhan et al., 2016). Here, we found that lncR-125b has 4,424 base pairs, located on chromosome 1 in goat, and has one potential binding site with chi-miR-125b, using the BLAST tool in the Ensembl database (Zerbino et al., 2018) (**Figure 1A** and **Figure S2**). Coding potential calculator (CPC) prediction showed that lncR-125b has very low coding potential, similar to that of XIST, a well-known lncRNA (Dimond and Fraser, 2013) (**Figure 1B**). Next, we examined its expression patterns in different tissues, including heart, liver, spleen, lung, kidney, and LTL muscle. We found that lncR-125b was predominantly expressed in LTL muscle (*P* < 0.05) (**Figure 1C**). Furthermore, we measured the expression of lncR-125b at different stages of goat SMSC differentiation (GM and DM for 1, 3, 5, and 7 d). The results showed that the expression of lncR-125b increased gradually during SMSC differentiation and reached a maximum in cells cultured in DM for 7 days (**Figure 1D**).

### lncR-125b Promotes SMSCs Differentiation

**Figure 2:**
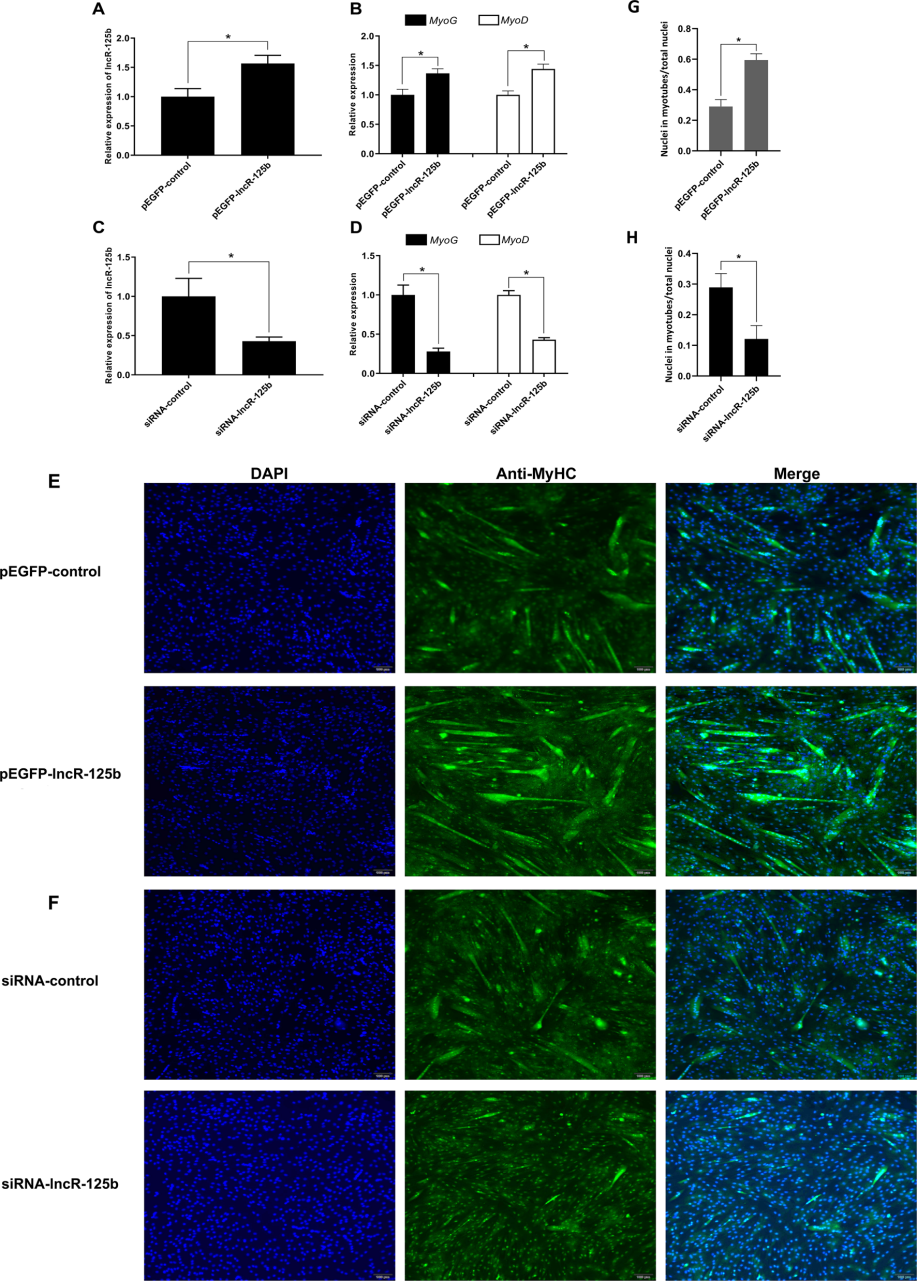
lncR-125b promotes myogenic differentiation *in vitro*. **(A)** Real-time PCR analysis of lncR-125b expression in SMSCs transfected with pEGFP-control or pEGFP-lncR-125b. **(B)** Real-time PCR analysis of MyoG and MyoD expression in SMSCs transfected with pEGFP-control or pEGFP-lncR-125b. **(C)** Real-time PCR analysis of lncR-125b expression in SMSCs transfected with siRNA-control or siRNA-lncR-125b. **(D)** Real-time PCR analysis of MyoG and MyoD expression in SMSCs transfected with siRNA-control or siRNA-lncR-125b. **(E)** MyHC immunostaining of SMSCs transfected with pEGFP-control vector or pEGFP-lncR-125b then cultured in differentiation medium (DM) for 7 days. The nuclei were visualized with DAPI (blue). Scale bar: 100 μm. **(F)** MyHC immunostaining of SMSCs transfected with siRNA-control or siRNA-lncR-125b then cultured in DM for 7 days. The nuclei were visualized with DAPI (blue). Scale bar: 100 μm. **(G)** Analysis of MyHC-staining cells after overexpression of lncR-125b. The percentage of MyHC positive cell was calculated as the ratio of the number of nuclei surrounded by MyHC signal to the total nuclei. **(H)** Analysis of MyHC-staining cells after knockdown of lncR-125b. The percentage of MyHC positive cell was calculated as the ratio of the number of nuclei surrounded by MyHC signal to the total nuclei. All data are shown as mean ± SEM of three biological replicates, *P < 0.05.

To explore the function of lncR-125b in the differentiation of SMSCs, we performed gain/loss-of-function experiments on lncR-125b in SMSCs. Firstly, we transfected SMSCs with pEGFP-lncR-125b (an expression vector overexpressing lncR-125b) or pEGFP-control, and siRNA-lncR-125b (an siRNA that knocks down lncR-125b expression) or siRNA-control. Overexpression of lncR-125b (**Figure 2A**) significantly upregulated the expression of myogenic marker genes MyoG and MyoD (**Figure 2B**), accelerated the differentiation of SMSCs with increased myosin heavy chain (MyHC) immunostaining (**Figure 2E**), and increased the number of myotubes with three or more nuclei (**Figure 2F**). Moreover, knockdown of lncR-125b (**Figure 2C**) significantly downregulated MyoG and MyoD expression (**Figure 2D**) and resulted in the inhibition of SMSC differentiation, as assessed by reduced MyHC immunostaining (**Figure 2F**) and a decreased number of myotubes with three or more nuclei (**Figure 2H**). In conclusion, these results demonstrate that lncR-125b can promote goat SMSC differentiation.

### lncR-125b Acts as a Molecular Sponge for miR-125b

**Figure 3:**
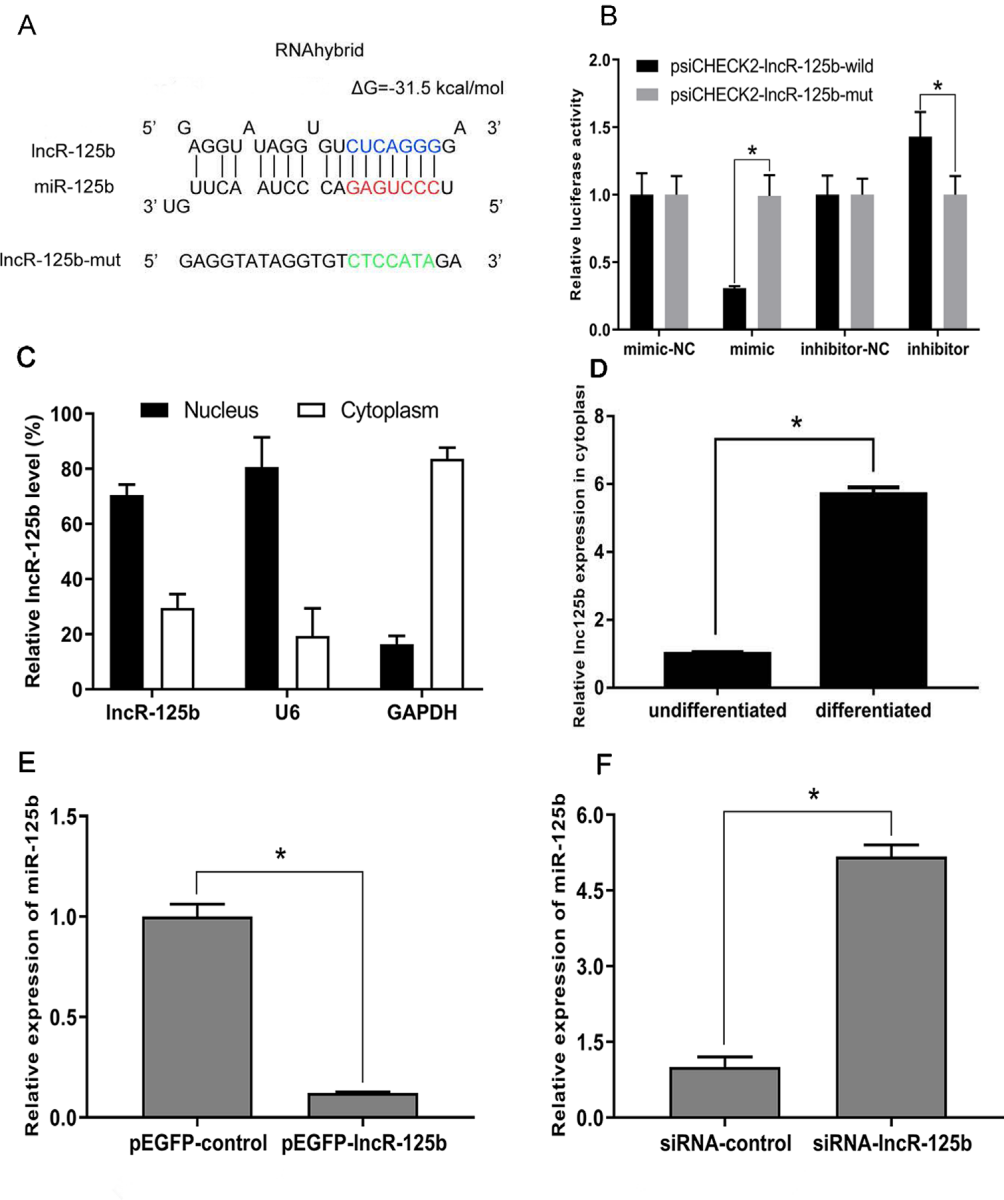
lncR-125b functions as a molecular sponge for miR-125b. **(A)** The miR-125b binding site in lncR-125b sequence was analyzed by RNAhybrid software. The seed sequence in miR-125b and mutant sequence in lncR-125b were highlighted in red and green, respectively. **(B)** The dual-luciferase reporter assay was performed by co-transfecting a lncR-125b-wild or lncR-125b-mut with miR-125b mimic or inhibitor in SMSCs. mimic-NC represents the miR-125b mimic negative control, and mimic represents the miR-125b mimic, inhibitor-NC represents the miR-125b inhibitor negative control, and inhibitor represents the miR-125b inhibitor. **(C)** The levels of lncR-125b were assessed by qRT-PCR in nuclear and cytoplasmic fractions. RNAs from nuclear/cytoplasmic fractionation of SMSCs were separately extracted GAPDH and U6 were used as cytoplasmic and nuclear markers, respectively. **(D)** Levels of lncR-125b in the cytoplasm of cultured proliferating (0 day) and differentiating (7 days) SMSCs. **(E)** Real-time PCR analysis of miR-125b expression in SMSCs transfected with pEGFP-control or pEGFP-lncR-125b. **(F)** Real-time PCR analysis of miR-125b expression in SMSCs transfected with siRNA-control or siRNA-lncR-125b. All data are given as mean ± SEM of three biological replicates, *P < 0.05.

To explore the mechanism underlying the activity of lncR-125b, we performed a bioinformatic analysis of the goat lncR-125b sequence and determined that it contains a potential miR-125b binding site (**Figure 3A**). To confirm that lncR-125b is indeed targeted by miR-125b, wild-type and mutant lncR-125b were synthesized, and luciferase reporters containing a wild-type or mutant target site from lncR-125b were also constructed. Our results showed that the relative luciferase activity of lncR-125b-wild was significantly reduced upon co-transfected with miR-125b mimic, compared with that in negative controls, while the down-regulation of miR-125b caused significant enhancement of relative luciferase activity (**Figure 3B**), suggesting the binding between lncR-125b and miR-125b. Based on these results, we speculated that lncR-125b may function as a ceRNA, leading to the liberation of corresponding miRNA-targeted transcripts. To test this hypothesis, we first determined the cellular location of lncR-125b. We partitioned the nuclear and cytosolic RNA from SMSCs. The results showed that lncR-125b localizes in both the cytoplasm and the nucleus (**Figure 3C**), and that the amount of lncR-125b in the cytoplasm increases significantly in differentiated SMSCs (**Figure 3D**). Next, we detected miR-125b expression in SMSCs with lncR-125b overexpression or lncR-125b knockdown. We determined that miR-125b was substantially downregulated when lncR-125b was overexpressed (**Figure 3E**), while it was considerably upregulated when lncR-125b was knocked down (**Figure 3F**). Taken together, all of these data demonstrate that lncR-125b contains a functional miR-125b binding site.

### IGF2 Is a Direct Target of miR-125b During Goat Myogenesis

**Figure 4:**
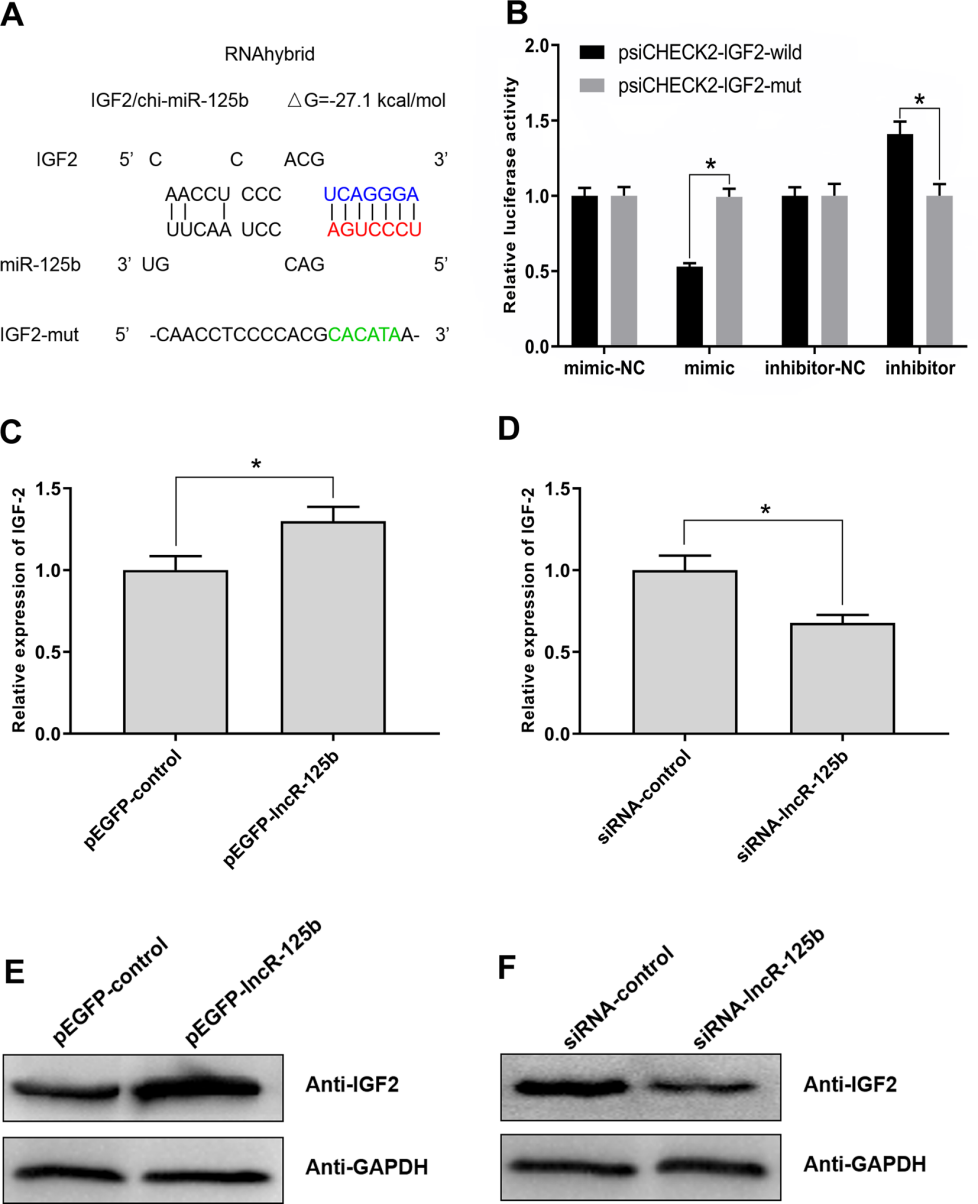
IGF2 is the target gene for miR-125b in goat and lncR-125b regulates the expression of IGF2. **(A)** The miR-125b binding site in IGF2 sequence was analyzed by RNAhybrid software. The seed sequence in miR-125b and mutant sequence in IGF2 were highlighted in red and green, respectively. **(B)** The dual-luciferase reporter assay was performed by co-transfecting a IGF2-wild or IGF2-mut with miR-125b mimic or inhibitor in SMSCs. mimic-NC represents the miR-125b mimic negative control, and mimic represents the miR-125b mimic, inhibitor-NC represents the miR-125b inhibitor negative control, and inhibitor represents the miR-125b inhibitor. **(C)** Real-time PCR analysis of IGF2 expression in SMSCs transfected with pEGFP-control or pEGFP-lncR-125b. **(D)** Real-time PCR analysis of IGF2 expression in SMSCs transfected with siRNA-control or siRNA-lncR-125b. **(E)** Western blot analysis of IGF2 protein abundances in SMSCs transfected with pEGFP-control or pEGFP-lncR-125b. **(F)** Western blot analysis of IGF2 protein abundances in SMSCs transfected with siRNA-control or siRNA-lncR-125b. All data are given as mean ± SEM of three biological replicates, *P < 0.05.

For further investigation of the possible downstream effectors of lncR-125b/miR-125b-mediated regulation of goat SMSC differentiation, we focused on IGF2, which was shown to act as a target of miR-125b and be regulated by miR-125b in mouse myoblast differentiation (Ge et al., 2011). Bioinformatic analysis of miRNA recognition sequences on goat IGF2 also indicated the presence of a putative miR-125b binding site (**Figure 4A**). To confirm the site of binding between IGF2 and miR-125b, we performed a luciferase reporter assay. The results revealed that the relative luciferase activity of IGF2-wild was decreased upon co-transfection with miR-125b mimic, compared with that in negative controls (**Figure 4B**). In contrast, the relative luciferase activity of IGF2-wild was increased upon co-transfection with miR-125b inhibitor (**Figure 4B**). These results confirmed that IGF2 is indeed a target of miR-125b in goat.

### lncR-125b Regulates the Expression of IGF2

**Figure 5:**
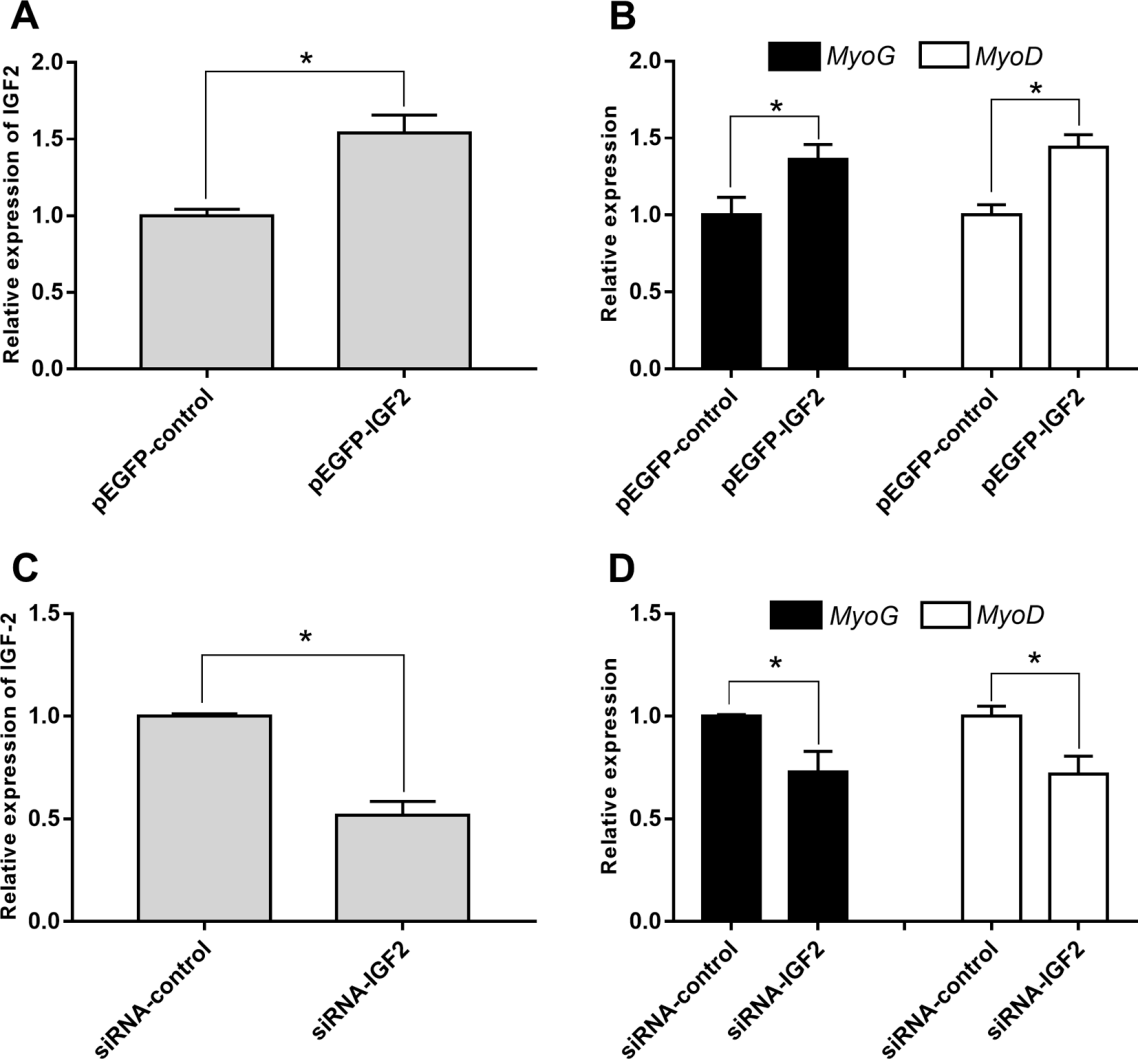
IGF2 promotes the differentiation of goat SMSCs. **(A)** Real-time PCR analysis of IGF2 expression in SMSCs transfected with pEGFP-control or pEGFP-IGF2. **(B)** Real-time PCR analysis of MyoG and MyoD expression in SMSCs transfected with pEGFP-control or pEGFP-IGF2. **(C)** Real-time PCR analysis of IGF2 expression in SMSCs transfected with siRNA-control or siRNA-IGF2. **(D)** Real-time PCR analysis of MyoG and MyoD expression in SMSCs transfected with siRNA-control or siRNA-IGF2. All data are given as mean ± SEM of three biological replicates, *P < 0.05.

To explore whether IGF2 was regulated by lncR-125b in goat SMSCs, we detected IGF2 mRNA and protein expression in SMSCs with lncR-125b overexpression or lncR-125b knockdown. The results indicated that both IGF2 mRNA and protein were significantly increased when lncR-125b was overexpressed (**Figures 4C, E**), while they were significantly decreased when lncR-125b was knocked down (**Figures 4D, F**). The expression of IGF2 is positively correlated with the expression level of lncR-125b. These results are consistent with the known mechanism of ceRNA, that lncR-125b competitively “sponges” miR-125b, leading to derepression of its target gene IGF2. Furthermore, to examine the role of IGF2 during skeletal myogenesis, we performed IGF2 gain/loss-of-function experiments in goat SMSCs. The results showed that overexpression of IGF2 (**Figure 5A**) significantly upregulated the expression of MyoG and MyoD (**Figure 5B**) and the knockdown of IGF2 (**Figure 5C**) significantly downregulated MyoG and MyoD expression (**Figure 5D**).

**Figure 6:**
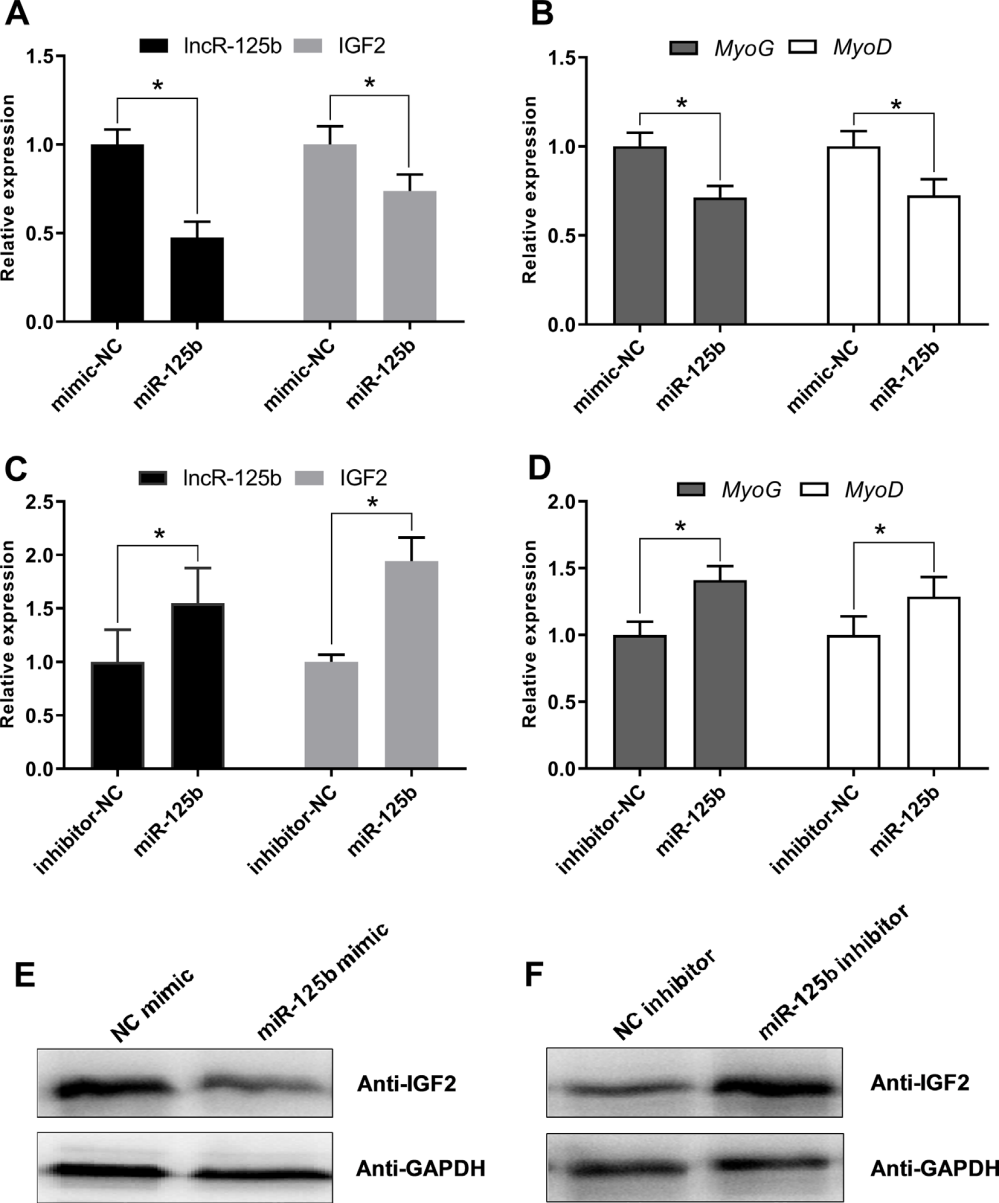
miR-125b negatively regulates SMSC differentiation and modulates the expression of lncR-125b and IGF2. **(A)** Real-time PCR analysis of lncR-125b and IGF2 expression in SMSCs transfected with miR-125b mimic and miR-125b mimic negative control. **(B)** Real-time PCR analysis of MyoG and MyoD expression in SMSCs transfected with miR-125b mimic and miR-125b mimic negative control. **(C)** Real-time PCR analysis of lncR-125b and IGF2 expression in SMSCs transfected with miR-125b inhibitor and miR-125b inhibitor negative control. **(D)** Real-time PCR analysis of MyoG and MyoD expression in SMSCs transfected with miR-125b inhibitor and miR-125b inhibitor negative control. **(E)** Western blot analysis of IGF2 protein expression in SMSCs transfected with miR-125b mimic and miR-125b mimic negative control. **(F)** Western blot analysis of IGF2 protein expression in SMSCs transfected with miR-125b inhibitor and miR-125b inhibitor negative control. All data are given as mean ± SEM of three biological replicates, *P < 0.05.

**Figure 7:**
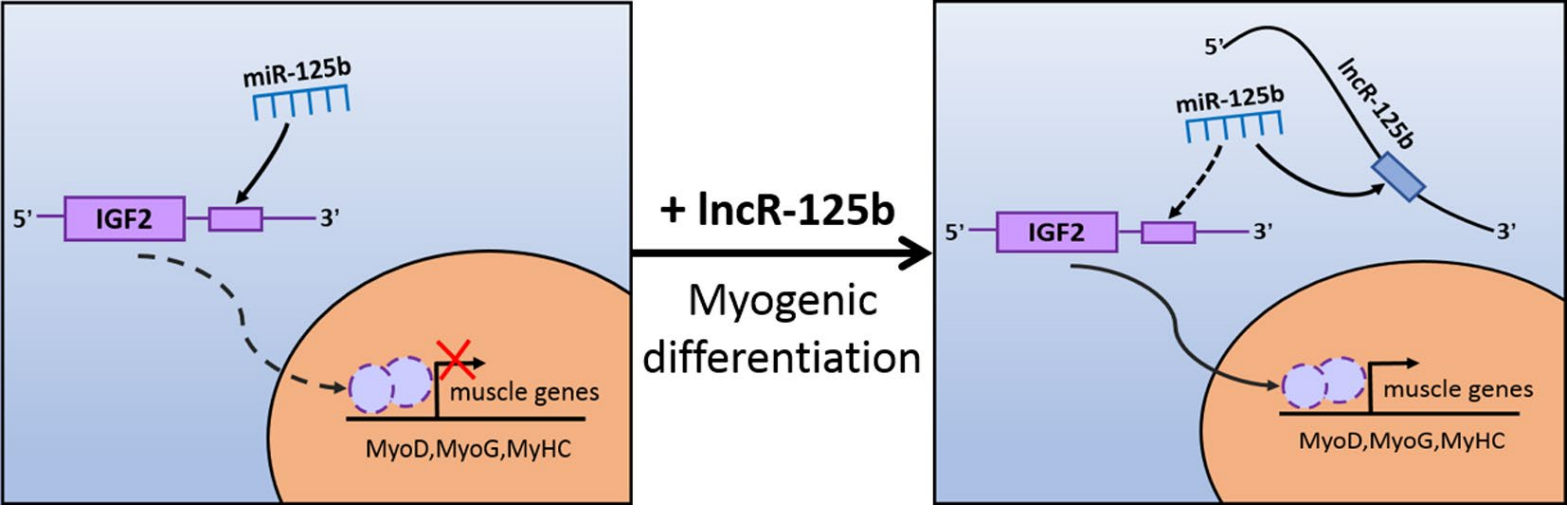
Schematic representation of how lncR-125b regulates myogenic differentiation. lncR-125b regulates myogenesis by functioning as a ceRNA that sequesters miR-125b, thereby relieving its repressive effect on IGF2 to promote muscle differentiation.

Moreover, to confirm that lncR-125b acts as a ceRNA for miR-125b from the opposite perspective, we detected the expression of lncR-125b, IGF2, and myogenic marker genes in goat SMSCs treated with miR-125b mimic or inhibitors. The results showed that the expression levels of lncR-125b and IGF2 were significantly reduced following the treatment of miR-125b mimic compared with that of miR-125b mimic negative controls (**Figure 6A**), whereas miR-125b inhibitors significantly increased the expression levels of lncR-125b or IGF2, compared with that for miR-125b inhibitor negative controls (**Figure 6C**). The change in expression of MyoG and MyoD in SMSCs treated with miR-125b mimic or inhibitor indicated that miR-125b negatively regulates goat SMSC differentiation (**Figures 6B, D**). Furthermore, the protein level of IGF2 was inhibited by miR-125b overexpression and upregulated after miR-125b knockdown (**Figures 6E, F**). Taken together, these results strongly suggest that lncR-125b may function as a ceRNA by binding miR-125b and acting as a decoy to abolish miR-125b repressing activity on IGF2, and thereby promotes goat SMSC differentiation (**Figure 7**).

## Discussion

lncRNAs exhibit diverse regulatory mechanisms in skeletal myogenesis. For example, a long noncoding RNA, lncMyoD, regulates skeletal muscle differentiation by blocking IGF2-mRNA-binding protein 2 (IMP2)-mediated mRNA translation (Gong et al., 2015). Another lncRNA, MyoD Upstream Noncoding (MUNC), is a pro-myogenic lncRNA that acts directly or indirectly on multiple promoters of MyoD to increase myogenic gene expression in skeletal myogenesis (Mueller et al., 2015). Moreover, the lncRNA H19 has vital trans-regulatory function in skeletal muscle differentiation and regeneration that is mediated by miR-675-3p and miR-675-5p encoded within H19 (Dey et al., 2014). Furthermore, a noncoding RNA required for myoblast proliferation, lncR-31, was shown to play a significant role in sustaining cell proliferation and in counteracting differentiation (Ballarino et al., 2015), the molecular mechanism of which has been revealed to involve lncR-31 stabilizing the YB-1 factor, thus allowing its positive effect on Rock1 mRNA translation and suppression of differentiation (Dimartino et al., 2018). Accumulating evidence suggests that lncRNAs play a critical role in skeletal myogenesis while highlighting the need to systematically identify lncRNAs altered in skeletal muscle development. So far, large numbers of lncRNAs have been detected in the muscle tissue of pig (Zhao et al., 2015; Gao et al., 2017; Zou et al., 2017), sheep (Ren et al., 2017), cattle (Billerey et al., 2014; Liu et al., 2017), chicken (Li et al., 2012a; Li et al., 2016b), and mouse (Zhang et al., 2014). Therefore, we also identified lncRNAs in fetal and postnatal goat skeletal muscle tissue using RNA-Seq in previous studies (Zhan et al., 2016), and these results provide a useful resource for further functional studies of the roles of lncRNAs in goat muscle formation.

In recent years, several studies have described that some lncRNAs can sequester miRNAs and therefore protect their target mRNAs from repression (Yan et al., 2015; Huang et al., 2016; Li et al., 2016a; Qu et al., 2016; Sun et al., 2016; Cai et al., 2017; Jin et al., 2017). For instance, lncRNA-MIAT was shown to function as a ceRNA and to form a feedback loop with vascular endothelial growth factor and miR-150-5p to regulate endothelial cell function (Yan et al., 2015). Another lncRNA, AK017368, was found to act as a ceRNA, sponging miR-30c to promote the proliferation of C2C12 cells cultured in GM and suppresses the differentiation of C2C12 cells cultured in DM (Liang et al., 2018). In addition, lnc-mg was shown to be a key myogenesis enhancer that acts by working as a ceRNA for miR-125b controlling the abundance of IGF2 protein in mouse (Zhu et al., 2017). Moreover, lnc-SMET antagonizes miR-125b to control IGF2 protein abundance and then enhances muscle differentiation in sheep (Wei et al., 2018). In the present study, we identified that lncR-125b was particularly highly enriched in skeletal muscle among different tissues (heart, liver, spleen, lung, kidney, and LTL muscle) of embryonic goat, and that its expression gradually increased during SMSC differentiation. These results indicate that lncR-125b probably has some function in skeletal muscle development. It is noteworthy that one of the characteristics of lncRNAs is tissue-specific expression (Tsoi et al., 2015; Bunch, 2018), which is consistent with our results. To identify the function of lncR-125b in SMSCs, we performed lncR-125b gain/loss-of-function experiments. The muscle differentiation marker gene (MyoG and MyoD) detection indicated that lncR-125b was a functional lncRNA during SMSC differentiation. lncR-125b overexpression in SMSCs increased SMSC differentiation, whereas lncR-125b knockdown led to a decrease in SMSC differentiation. These results further suggest that lncR-125b plays a vital role in muscle differentiation. Subsequent bioinformatic analyses and dual-luciferase reporter gene assays indicated that lncR-125b is a direct target of miR-125b. Further miR-125b overexpression or knockdown experiments indicated that miR-125b negatively regulates goat SMSC differentiation, which is consistent with the findings in mice (Ge et al., 2011). Therefore, we speculate that lncR-125b may act as a ceRNA in the regulation of goat muscle differentiation. Interestingly, lncR-125b upregulation and downregulation negatively regulated miR-125b expression. These results further demonstrate that lncR-125b “sponges” miR-125b to accelerate goat SMSC differentiation. Conversely, miR-125b overexpression or knockdown negatively regulated lncR-125b expression. These results indirectly demonstrate that miRNA is involved in the regulation of lncRNA. A similar mechanism was described for MALAT1/miR-9 pathways involving Ago2-dependent lncRNA degradation, which reveal a novel direct regulatory link between miRNAs and lncRNAs (Leucci et al., 2013).

In search of the possible downstream effector of lncR-125b/miR-125b mediated regulation of SMSC differentiation, we focused on IGF2, a critical regulator of skeletal myogenesis (Stewart et al., 1996; Stewart and Rotwein, 1996; Ge et al., 2011; Jiao et al., 2013). IGF2, an embryonic regulator of myogenesis and an autocrine factor that initiates myoblast differentiation *in vitro* (Duan et al., 2010), is regulated at the transcriptional level through a muscle-specific enhancer by mammalian target of rapamycin (mTOR) signaling (Erbay et al., 2003; Ge and Chen, 2012). In accordance with the obtained results, the overexpression and knockdown of IGF2 in the present study revealed that it can promote the differentiation of goat SMSCs. Additionally, another study found that miR-125b negatively modulates myoblast differentiation *in vitro* and muscle regeneration *in vivo*, and IGF2 is the target gene of miR-125b in skeletal myogenesis (Ge et al., 2011). Thus, we performed dual-luciferase reporter assays to verify the binding relationship between IGF2 and miR-125b in goat. Mutation of the seed region of the predicted miR-125b binding site suppressed the regulation of the IGF2 3’ UTR reporter, demonstrating that IGF2 is a direct target of miR-125b in goat. Further investigation revealed that the lncR-125b expression was negatively and positively correlated with miR-125b and IGF2 expression levels during goat SMSC differentiation, and that lncR-125b regulates the expression of miR-125b and IGF2 in a dose-dependent manner (**Figure S3**), which is a crucial confirmation to the ceRNA function of lncR-125b.

In summary, we revealed that a muscle-specific lncRNA, lncR-125b, functions as a ceRNA to promote the differentiation of goat SMSCs by regulating the expression of the target gene of miR-125b. The lncR-125b/miR-125b/IGF2 regulatory network preliminarily explains the mechanisms of action of lncR-125b during skeletal myogenesis, providing new insight into the downstream mechanisms of lncRNAs regulating the differentiation of goat SMSCs.

## Data Availability Statement

All datasets supporting the conclusions of this study can be found in the article/**Supplementary Material**.

## Ethics Statement

All experimental procedures involving animals were conducted under a protocol approved by the Institutional Animal Care and Use Committee of the College of Animal Science and Technology, Sichuan Agricultural University under permit No.DKYB20110807. All animals were sacrificed humanely to minimize suffering.

## Author Contributions

SZ conceived and designed the experiments and wrote the paper. CQ, DL, WZ, and LN performed part of the experiments. JC and JG conducted the animal experiments and sampling. TZ and LW participated in the sampling and data analysis. LL and HZ provided the experimental environment and edited the manuscript. All authors have read and approved the final manuscript.

## Funding

The work was supported by the National key Research and Development Program of China (2018YFD0502002), National Natural Science Foundation of China (31672402, 31772578), Sichuan Science and Technology Program (2019YJ0429), and Independent Research Project of Farm Animal Genetic Resources Exploration and Innovation Key Laboratory of Sichuan Province (SNDK-ZZ-201702).

## Conflict of Interest

The authors declare that the research was conducted in the absence of any commercial or financial relationships that could be construed as a potential conflict of interest.

## Supplementary Material

Supplementary Figure 1 The sequences of wild-type and mutated sequences of lncR-125b and IGF2, and the binding site of miR-125b is marked in red.

Supplementary Figure 2 The Sequence of goat lncR-125b, where the binding site of miR-125b is marked in red.

Supplementary Figure 3 Expression patterns of lncR-125b, miR-125b, and IGF2 in SMSCs. **(A)** The real-time PCR analysis of lncR-125b, miR-125b and IGF2 in SMSCs after transfected with 2 μg, 4 μg or 6 μg lncR-125b expression vector for 48 h; **(B)** Real-time PCR analysis of the expression of lncR-125b, miR-125b, and IGF2 during goat SMSC differentiation (GM and DM for 1, 3, 5, and 7 d). All data are shown as mean ± SEM of three biological replicates.

Supplementary Table 1 Specific primers used for qRT-PCR.

Supplementary Table 2 Primers used for plasmid construction.
