# Supplementary figures and images for: A Novel Long Noncoding RNA, lncR-125b, Promotes the Differentiation of Goat Skeletal Muscle Satellite Cells by Sponging miR-125b
Source: Front Genet. 2019 Nov 15;10:1171. doi: 10.3389/fgene.2019.01171 (PMC6872680; doi:10.3389/fgene.2019.01171)

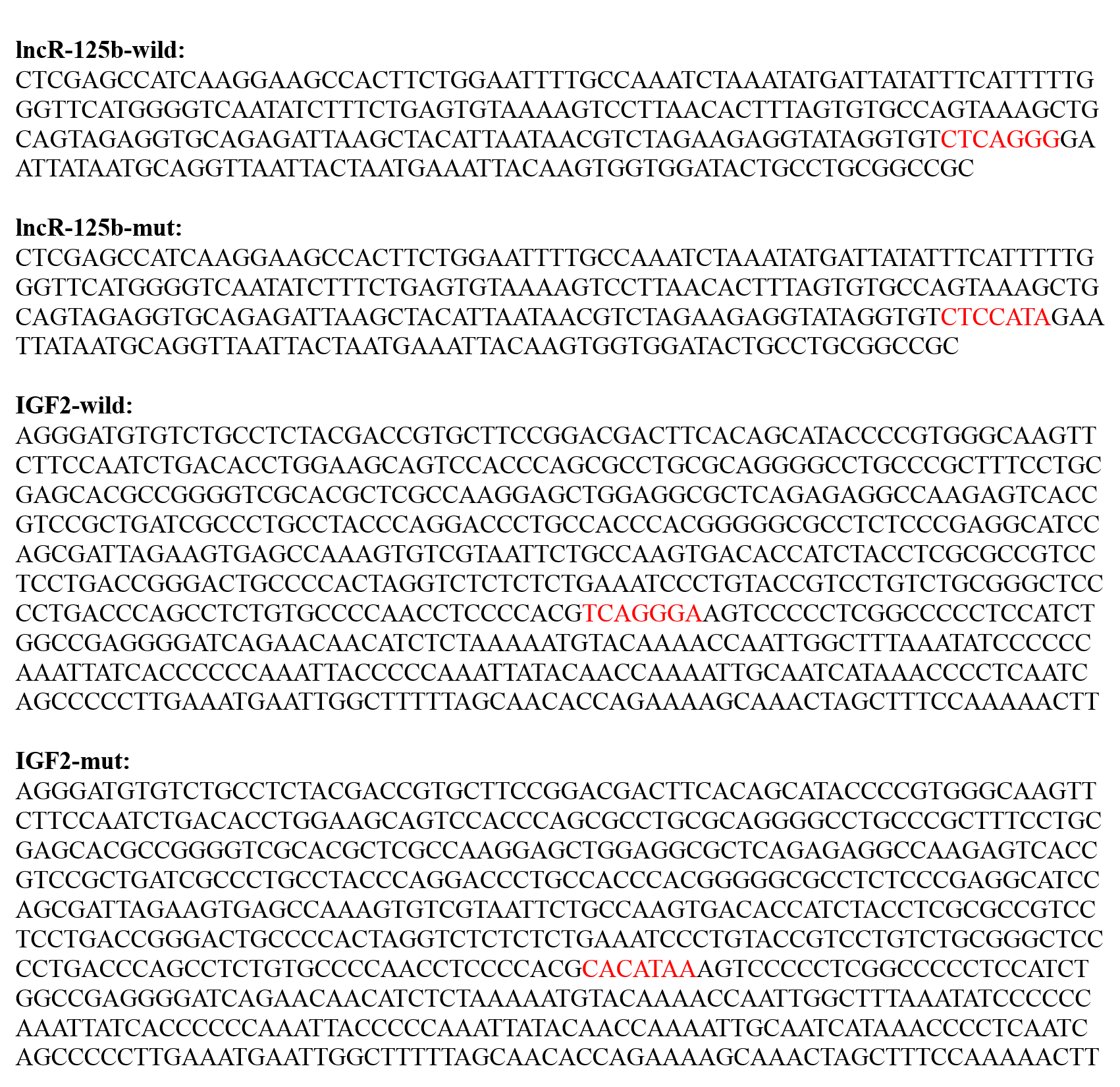

Supplement: Supplementary Figure 1 — The sequences of wild-type and mutated sequences of lncR-125b and IGF2, and the binding site of miR-125b is marked in red. [file Image_1.png]

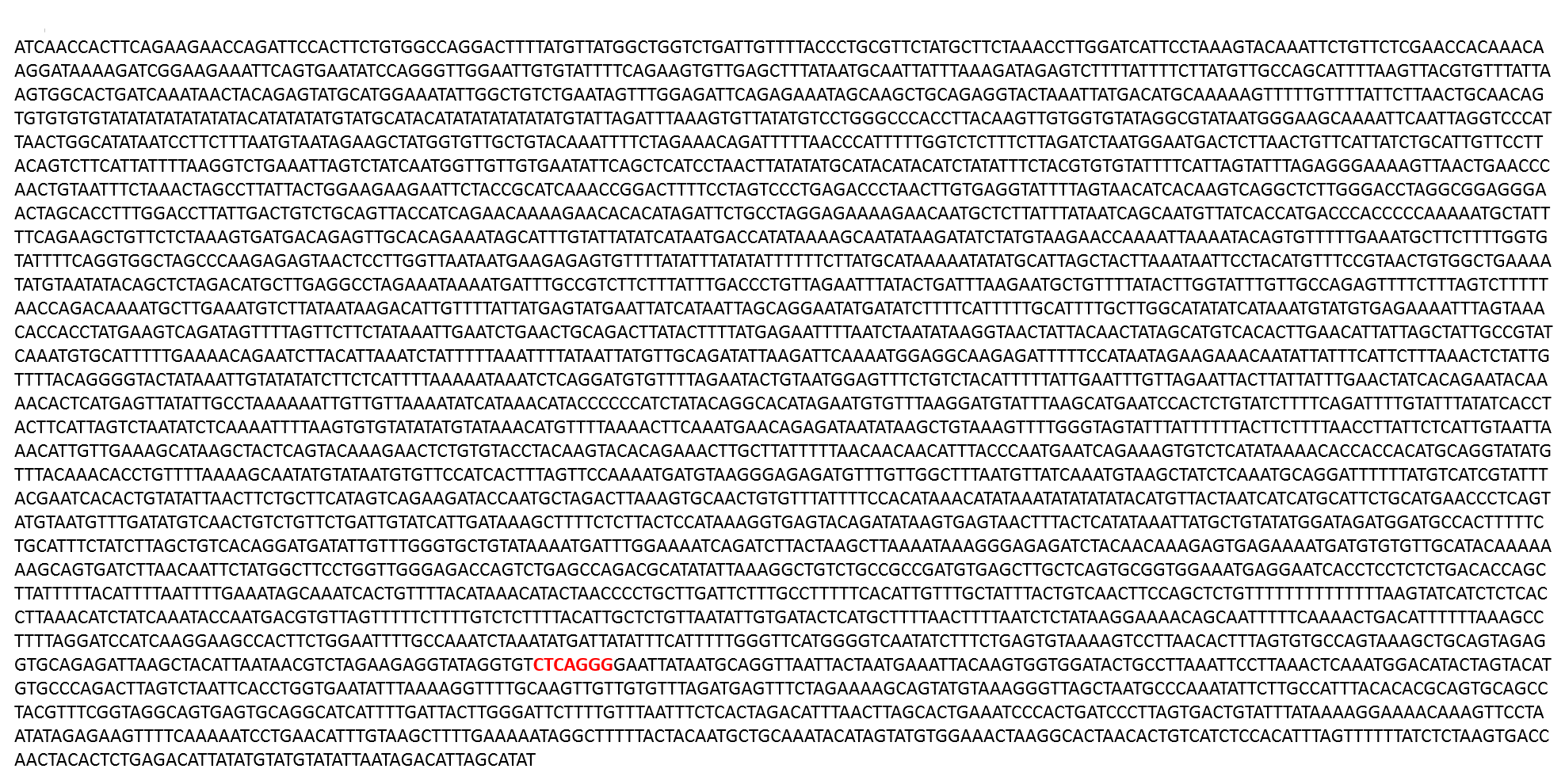

Supplement: Supplementary Figure 2 — The Sequence of goat lncR-125b, where the binding site of miR-125b is marked in red. [file Image_2.tif]

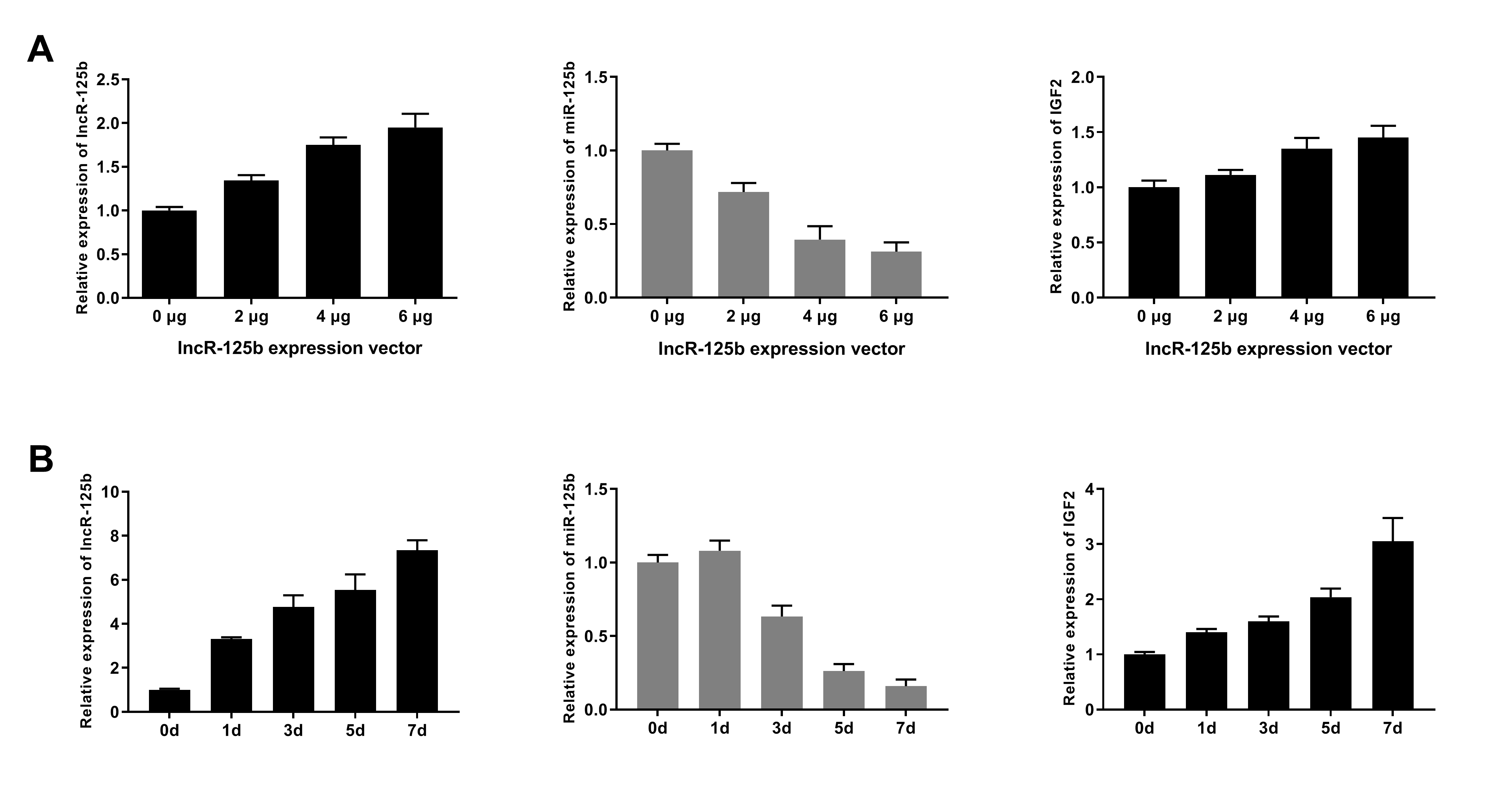

Supplement: Supplementary Figure 3 — Expression patterns of lncR-125b, miR-125b, and IGF2 in SMSCs. (A) The real-time PCR analysis of lncR-125b, miR-125b and IGF2 in SMSCs after transfected with 2 μg, 4 μg or 6 μg lncR-125b expression vector for 48 h; (B) Real-time PCR analysis of the expression of lncR-125b, miR-125b, and IGF2 during goat SMSC differentiation (GM and DM for 1, 3, 5, and 7 d). All data are shown as mean ± SEM of three biological replicates. [file Image_3.tif]
