# Supplementary material for: A Novel Long Noncoding RNA, lncR-125b, Promotes the Differentiation of Goat Skeletal Muscle Satellite Cells by Sponging miR-125b
Source: Front Genet. 2019 Nov 15;10:1171. doi: 10.3389/fgene.2019.01171 (PMC6872680; doi:10.3389/fgene.2019.01171)
Supplement: Supplementary file 6 [file DataSheet_1.pdf]

Supplementary Material

1 Supplementary Figures and Tables

1.1 Supplementary Figures

lncR-125b-wild:
CTCGAGCCATCAAGGAAGCCACTTCTGGAATTTTGCCAAATCTAAATATGATTATATTTCATTTTTG
GGTTCATGGGGTCAATATCTTTCTGAGTGTAAAAGTCCTTAACACTTTAGTGTGCCAGTAAAGCTG
CAGTAGAGGTGCAGAGATTAAGCTACATTAATAACGTCTAGAAGAGGTATAGGTGTCTCAGGGGA
ATTATAATGCAGGTTAATTACTAATGAAATTACAAGTGGTGGATACTGCCTGCGGCCGC

lncR-125b-mut:
CTCGAGCCATCAAGGAAGCCACTTCTGGAATTTTGCCAAATCTAAATATGATTATATTTCATTTTTG
GGTTCATGGGGTCAATATCTTTCTGAGTGTAAAAGTCCTTAACACTTTAGTGTGCCAGTAAAGCTG
CAGTAGAGGTGCAGAGATTAAGCTACATTAATAACGTCTAGAAGAGGTATAGGTGTCTCCATAGAA
TTATAATGCAGGTTAATTACTAATGAAATTACAAGTGGTGGATACTGCCTGCGGCCGC

IGF2-wild:
AGGGATGTGTCTGCCTCTACGACCGTGCTTCCGGACGACTTCACAGCATACCCCGTGGGCAAGTT
CTTCCAATCTGACACCTGGAAGCAGTCCACCCAGCGCCTGCGCAGGGGCCTGCCCGCTTTCCTGC
GAGCACGCCGGGGTCGCACGCTCGCCAAGGAGCTGGAGGCGCTCAGAGAGGCCAAGAGTCACC
GTCCGCTGATCGCCCTGCCTACCCAGGACCCTGCCACCCACGGGGGCGCCTCTCCCGAGGCATCC
AGCGATTAGAAGTGAGCCAAAGTGTCGTAATTCTGCCAAGTGACACCATCTACCTCGCGCCGTCC
TCCTGACCGGGACTGCCCCACTAGGTCTCTCTCTGAAATCCCTGTACCGTCCTGTCTGCGGGCTCC
CCTGACCCAGCCTCTGTGCCCCAACCTCCCCACGTCAGGGAAGTCCCCCTCGGCCCCCTCCATCT
GGCCGAGGGGATCAGAACAACATCTCTAAAAATGTACAAAACCAATTGGCTTTAAATATCCCCCC
AAATTATCACCCCCCAAATTACCCCCAAATTATACAACCAAAATTGCAATCATAAACCCCTCAATC
AGCCCCCTTGAAATGAATTGGCTTTTTAGCAACACCAGAAAAGCAAACTAGCTTTCCAAAAACTT

IGF2-mut:
AGGGATGTGTCTGCCTCTACGACCGTGCTTCCGGACGACTTCACAGCATACCCCGTGGGCAAGTT
CTTCCAATCTGACACCTGGAAGCAGTCCACCCAGCGCCTGCGCAGGGGCCTGCCCGCTTTCCTGC
GAGCACGCCGGGGTCGCACGCTCGCCAAGGAGCTGGAGGCGCTCAGAGAGGCCAAGAGTCACC
GTCCGCTGATCGCCCTGCCTACCCAGGACCCTGCCACCCACGGGGGCGCCTCTCCCGAGGCATCC
AGCGATTAGAAGTGAGCCAAAGTGTCGTAATTCTGCCAAGTGACACCATCTACCTCGCGCCGTCC
TCCTGACCGGGACTGCCCCACTAGGTCTCTCTCTGAAATCCCTGTACCGTCCTGTCTGCGGGCTCC
CCTGACCCAGCCTCTGTGCCCCAACCTCCCCACGCACATAAAGTCCCCCTCGGCCCCCTCCATCT
GGCCGAGGGGATCAGAACAACATCTCTAAAAATGTACAAAACCAATTGGCTTTAAATATCCCCCC
AAATTATCACCCCCCAAATTACCCCCAAATTATACAACCAAAATTGCAATCATAAACCCCTCAATC
AGCCCCCTTGAAATGAATTGGCTTTTTAGCAACACCAGAAAAGCAAACTAGCTTTCCAAAAACTT

Supplementary Figure 1. The sequences of wild-type and mutated sequences of lncR-125b and IGF2, and the binding site of miR-125b is marked in red.

ATCAACCACTTCAGAAGAACCAGATTCCACTTCTGTGGCCAGGACTTTTATGTTATGGCTGGTCTGATTGTTTTACCCTGCGTTCTATGCTTCTAAACCTTGGATCATTCCTAAAGTACAAATTCTGTTCTCGAACCACAAACA
AGGATAAAAGATCGGAAGAAATTCAGTGAATATCCAGGGTTGGAATTGTGTATTTTCAGAAGTGTTGAGCTTTATAATGCAATTATTTAAAGATAGAGTCTTTTATTTTCTTATGTTGCCAGCATTTTAAGTTACGTGTTTATTA
AGTGGCACTGATCAAATAACTACAGAGTATGCATGGAAATATTGGCTGTCTGAATAGTTTGGAGATTCAGAGAAATAGCAAGCTGCAGAGGTACTAAATTATGACATGCAAAAAGTTTTTGTTTTATTCTTAACTGCAACAG
TGTGTGTGTATATATATATATATACATATATATGTATGCATACATATATATATATATGTATTAGATTTAAAGTGTTATATGTCCTGGGCCCACCTTACAAGTTGTGGTGTATAGGCGTATAATGGGAAGCAAAATTCAATTAGGTCCCAT
TAACTGGCATATAATCCTTCTTTAATGTAATAGAAGCTATGGTGTTGCTGTACAAATTTTCTAGAAACAGATTTTTAACCCATTTTTGGTCTCTTTCTTAGATCTAATGGAATGACTCTTAACTGTTCATTATCTGCATTGTTCCTT
ACAGTCTTCATTATTTTAAGGTCTGAAATTAGTCTATCAATGGTTGTTGTGAATATTCAGCTCATCCTAACTTATATATGCATACATACATCTATATTTCTACGTGTGTATTTTCATTAGTATTTAGAGGGAAAAGTTAACTGAACCC
AACTGTAATTTCTAAACTAGCCTTATTACTGGAAGAAGAATTCTACCGCATCAAACCGGACTTTTCCTAGTCCCTGAGACCCTAACTTGTGAGGTATTTTAGTAACATCACAAGTCAGGCTCTTGGGACCTAGGCGGAGGGA
ACTAGCACCTTTGGACCTTATTGACTGTCTGCAGTTACCATCAGAACAAAAGAACACACATAGATTCTGCCTAGGAGAAAAGAACAATGCTCTTATTTATAATCAGCAATGTTATCACCATGACCCACCCCCAAAAATGCTATT
TTCAGAAGCTGTTCTCTAAAGTGATGACAGAGTTGCACAGAAATAGCATTTGTATTATATCATAATGACCATATAAAAGCAATATAAGATATCTATGTAAGAACCAAAATTAAAATACAGTGTTTTTGAAATGCTTCTTTTGGTG
TATTTTCAGGTGGCTAGCCCAAGAGAGTAACTCCTTGGTTAATAATGAAGAGAGTGTTTTATATTTATATATTTTTTCTTATGCATAAAAATATATGCATTAGCTACTTAAATAATTCCTACATGTTTCCGTAACTGTGGCTGAAAA
TATGTAATATACAGCTCTAGACATGCTTGAGGCCTAGAAATAAAATGATTTGCCGTCTTCTTTATTTGACCCTGTTAGAATTTATACTGATTTAAGAATGCTGTTTTATACTTGGTATTTGTTGCCAGAGTTTTCTTTAGTCTTTTT
AACCAGACAAAATGCTTGAAATGTCTTATAATAAGACATTGTTTTATTATGAGTATGAATTATCATAATTAGCAGGAATATGATATCTTTTCATTTTTGCATTTTGCTTGGCATATATCATAAATGTATGTGAGAAAATTTAGTAAA
CACCACCTATGAAGTCAGATAGTTTTAGTTCTTCTATAAATTGAATCTGAACTGCAGACTTATACTTTTATGAGAATTTTAATCTAATATAAGGTAACTATTACAACTATAGCATGTCACACTTGAACATTATTAGCTATTGCCGTAT
CAAATGTGCATTTTTGAAAACAGAATCTTACATTAAATCTATTTTTAAATTTTATAATTATGTTGCAGATATTAAGATTCAAAATGGAGGCAAGAGATTTTTCCATAATAGAAGAAACAATATTATTTCATTCTTTAAACTCTATTG
TTTTACAGGGGTACTATAAATTGTATATATCTTCTCATTTTAAAAATAAATCTCAGGATGTGTTTTAGAATACTGTAATGGAGTTTCTGTCTACATTTTTATTGAATTTGTTAGAATTACTTATTATTTGAACTATCACAGAATACAA
AACACTCATGAGTTATATTGCCTAAAAAATTGTTGTTAAAATATCATAAACATACCCCCCATCTATACAGGCACATAGAATGTGTTTAAGGATGTATTTAAGCATGAATCCACTCTGTATCTTTTCAGATTTTGTATTTATATCACCT
ACTTCATTAGTCTAATATCTCAAAATTTTAAGTGTGTATATATGTATAAACATGTTTTAAAACTTCAAATGAACAGAGATAATATAAGCTGTAAAGTTTTGGGTAGTATTTATTTTTTACTTCTTTTAACCTTATTCTCATTGTAATTA
AACATTGTTGAAAGCATAAGCTACTCAGTACAAAGAACTCTGTGTACCTACAAGTACACAGAAACTTGCTTATTTTTAACAACAACATTTACCCAATGAATCAGAAAGTGTCTCATATAAAACACCACCACATGCAGGTATATG
TTTACAAACACCTGTTTTAAAAGCAATATGTATAATGTGTTCCATCACTTTAGTTCCAAAATGATGTAAGGGAGAGATGTTTGTTGGCTTTAATGTTATCAAATGTAAGCTATCTCAAATGCAGGATTTTTTATGTCATCGTATTT
ACGAATCACACTGTATATTAACTTCTGCTTCATAGTCAGAAGATACCAATGCTAGACTTAAAGTGCAACTGTGTTTATTTTCCACATAAACATATAAATATATATATACATGTTACTAATCATCATGCATTCTGCATGAACCCTCAGT
ATGTAATGTTTGATATGTCAACTGTCTGTTCTGATTGTATCATTGATAAAGCTTTTCTCTTACTCCATAAAGGTGAGTACAGATATAAGTGAGTAACTTTACTCATATAAATTATGCTGTATATGGATAGATGGATGCCACTTTTTC
TGCATTTCTATCTTAGCTGTCACAGGATGATATTGTTTGGGTGCTGTATAAAATGATTTGGAAAATCAGATCTTACTAAGCTTAAAATAAAGGGAGAGATCTACAACAAAGAGTGAGAAAATGATGTGTGTTGCATACAAAAA
AAGCAGTGATCTTAACAATTCTATGGCTTCCTGGTTGGGAGACCAGTCTGAGCCAGACGCATATATTAAAGGCTGTCTGCCGCCGATGTGAGCTTGCTCAGTGCGGTGGAAATGAGGAATCACCTCCTCTCTGACACCAGC
TTATTTTTACATTTTAATTTTGAAATAGCAAATCACTGTTTTACATAAACATACTAACCCCTGCTTGATTCTTTGCCTTTTTCACATTGTTTGCTATTTACTGTCAACTTCCAGCTCTGTTTTTTTTTTTTTTAAGTATCATCTCTCAC
CTTAAACATCTATCAAATACCAATGACGTGTTAGTTTTTCTTTTGTCTCTTTTACATTGCTCTGTTAATATTGTGATACTCATGCTTTTAACTTTTAATCTCTATAAGGAAAACAGCAATTTTTCAAAACTGACATTTTTTAAAGCC
TTTTAGGATCCATCAAGGAAGCCACTTCTGGAATTTTGCCAAATCTAAATATGATTATATTTCATTTTTGGGTTCATGGGGTCAATATCTTTCTGAGTGTAAAAGTCCTTAACACTTTAGTGTGCCAGTAAAGCTGCAGTAGAG
GTGCAGAGATTAAGCTACATTAATAACGTCTAGAAGAGGTATAGGTGT**CTCAGGG**GAATTATAATGCAGGTTAATTACTAATGAAATTACAAGTGGTGGATACTGCCTTAAATTCCTTAAACTCAAATGGACATACTAGTACAT
GTGCCCAGACTTAGTCTAATTCACCTGGTGAATATTTAAAAGGTTTTGCAAGTTGTTGTGTTTAGATGAGTTTCTAGAAAAGCAGTATGTAAAGGGTTAGCTAATGCCCAAATATTCTTGCCATTTACACACGCAGTGCAGCC
TACGTTTCGGTAGGCAGTGAGTGCAGGCATCATTTTGATTACTTGGGATTCTTTTGTTTAATTTCTCACTAGACATTTAACTTAGCACTGAAATCCCACTGATCCCTTAGTGACTGTATTTATAAAAGGAAAACAAAGTTCCTA
ATATAGAGAAGTTTTCAAAAATCCTGAACATTTGTAAGCTTTTGAAAAATAGGCTTTTTACTACAATGCTGCAAATACATAGTATGTGGAAACTAAGGCACTAACACTGTCATCTCCACATTTAGTTTTTTATCTCTAAGTGACC
AACTACACTCTGAGACATTATATGTATGTATATTAATAGACATTAGCATAT

Supplementary Figure 2. The Sequence of goat lncR-125b, where the binding site of miR-125b is marked in red.

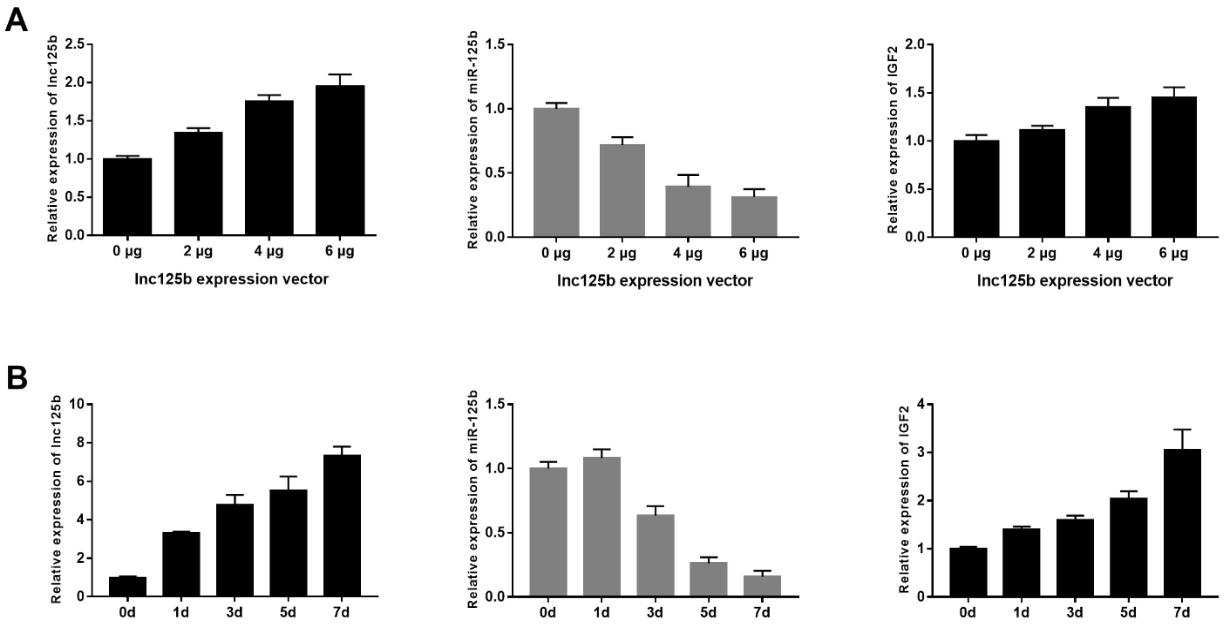

Supplementary Figure 3. Expression patterns of lncR-125b, miR-125b, and IGF2 in SMSCs. **(A)** The real-time PCR analysis of lncR-125b, miR-125b and IGF2 in SMSCs after transfected with 2 μg, 4 μg or 6 μg lncR-125b expression vector for 48 h; **(B)** Real-time PCR analysis of the expression of lncR-

125b, miR-125b, and IGF2 during goat SMSC differentiation (GM and DM for 1, 3, 5, and 7 d). All data are shown as mean ± SEM of three biological replicates.

1.2 Supplementary Tables

Table S1. Specific primers used for qRT-PCR.

Primer Name	Primer Sequences (5’ to 3’)	Tm. (℃)	Size (bp)
lncR-125b	F: AGCAGCAGTGAGCGTGAGAT<br>R: TGGCAGCAGAATGGTAGAAGGT	58.3	161
IGF2	F: TCGTGCTGCTATGCTGCTTAC<br>R: TGGATGGTCGGCTGAAGTAGAA	61.4	112
MyoG	F: CGCAGACTCAAGAAGGTGAA<br>R: CGCTCTATGTACTGGATGGC	59.7	119
MyoD	F: GTGCAAACGCAAGACGACTA<br>R: GCTGGTTTGGGTTGCTAGAC	59.7	128
ACTB	F: CCTGCGGCATTCACGAAACTAC<br>R: ACAGCACCGTGTTGGCGTAGAG	59.7	87
YWHAZ	F: TACTATCGCTACTTGGCTGAGG<br>R: GCTTCTTGGTATGCTTGCTGTG	61.4	83
HPRT1	F: CAGCGTGGTGATTAGCGATGAT<br>R: AGTCGTTCGGTCCTGTCCATAA	61.4	129
GAPDH	F: GCAAGTTCCACGGCACAG<br>R: GGTTCACGCCCATCACAA	59	249
miR-125b	F: TCCCTGAGACCCTAACTTGT	60	
U6	F: CAAGGATGACACGCAAATTCG	59	

Table S2. Primers used for plasmid construction.

Primer Name	Primer Sequences (5’ to 3’)
pEGFP-lncR-125b	F: CCC**AAGCTT**CCATCAAGGAAGCCACTT<br>R: CG**GGATCC**AGGCAGTATCCACCACTT
pEGFP-IGF2	F: CCC**AAGCTT**AGGGATGTGTCTGCCTCTAC<br>R: CG**GGATCC**AAGTTTTTGGAAAGCTAGTT

lncR-125b-wild	F: CCG**CTCGAG**GGTGTCTCAGGGGAATTATA
	R: AAATAT**GCGGCCGC**TATACCTCTTCTAGACGTTA
lncR-125b-mut	F: CCG**CTCGAG**GGTGTCTCCATAGAATTATA
	R: AAATAT**GCGGCCGC**TATACCTCTTCTAGACGTTA
IGF2-wild	F: CCG**CTCGAG**TCCCCACGTCAGGGAAGTCC
	R: AAATAT**GCGGCCGC**AAGTTTTTGGAAAGCTAGTT
IGF2-mut	F: CCG**CTCGAG**TCCCCACGCACATAAAGTCC
	R: AAATAT**GCGGCCGC**AAGTTTTTGGAAAGCTAGTT

Underlined sequences represents the protected base. Sequences in bold represent the enzyme cutting sites.
